# Diagnostic Value of Inflammatory Biomarkers in Intracranial Venous Thrombosis: A Multi-model Predictive Analysis

**DOI:** 10.7759/cureus.74070

**Published:** 2024-11-20

**Authors:** Longmin Zhou, Wenting Jiang, Pengwei Hou, Mingfa Cai, Ziqi Li, Shousen Wang

**Affiliations:** 1 Department of Neurosurgery, Fuzong Clinical Medical College of Fujian Medical University (The 900th Hospital), Fuzhou, CHN; 2 Department of Neurosurgery, School of Public Health, Shenyang Medical College, Shenyang, CHN; 3 Department of Neurosurgery, Jinjiang Municipal Hospital (Shanghai Sixth People's Hospital), Jinjiang, CHN

**Keywords:** artificial neural network, cerebral venous thrombosis, inflammatory biomarkers, machine learning, systemic immune-inflammatory index

## Abstract

Objective: Cerebral venous thrombosis (CVT) is a rare but significant condition, primarily affecting young adults, especially women. The diagnosis of CVT is challenging due to its nonspecific clinical presentation. Inflammatory biomarkers, such as the systemic immune-inflammatory index (SII), platelet-to-lymphocyte ratio (PLR), and neutrophil-to-lymphocyte ratio (NLR), may aid in early diagnosis. This study aimed to explore the role of these biomarkers and assess machine learning models for improving diagnostic accuracy.

Methods: This study included 100 CVT patients and 50 controls. Data collected included demographic information, biochemical markers, and clinical symptoms. Traditional statistical methods and machine learning models, including decision trees, random forests, AdaBoost, k-nearest neighbors, support vector machines (SVM), and artificial neural networks (ANN), were used to evaluate the diagnostic value of biomarkers.

Results: The SII and NLR levels were significantly higher in CVT patients. The ANN model based on SII and PLR achieved the best diagnostic performance, with an area under the curve (AUC) of 0.94, showing high accuracy and reliability.

Conclusion: Inflammatory biomarkers, particularly SII, have significant predictive value in CVT diagnosis. Machine learning models, especially ANN, show promise in improving diagnostic accuracy. Future studies with larger sample sizes are needed to validate these findings further.

## Introduction

As a subtype of stroke, cerebral venous thrombosis (CVT) accounts for 0.5%-1% of all stroke cases [1]. Though relatively rare, it poses a significant health threat, particularly to young adults, especially women. Despite its low prevalence, CVT has a profound impact on patients and their families [2]. With advancements in medical technology and an increased awareness of health, the diagnostic rate of CVT has risen, reflecting improved medical understanding and diagnostic capabilities related to this condition.

The diagnosis and treatment of CVT present significant challenges due to its diverse risk factors, which include both inflammatory factors (such as infections and nonspecific inflammation) and non-inflammatory factors (such as hypercoagulability, blood stasis, vascular wall injury, and intracranial hypotension) [3, 4]. In recent years, the link between inflammation and CVT has garnered increasing attention, with studies indicating that specific inflammatory biomarkers play a crucial role in the diagnosis and prognosis of CVT [5]. Inflammatory markers such as the neutrophil-to-lymphocyte ratio (NLR), platelet-to-lymphocyte ratio (PLR), and systemic immune-inflammatory index (SII) have become focal points of research due to their objectivity, low cost, efficacy, and reproducibility [6-9]. Furthermore, modern magnetic resonance black-blood thrombus imaging (MRBTI) has demonstrated unique advantages in the visualization of thrombosis, providing a more intuitive tool for the diagnosis of CVT [10].

The relationship between inflammation and thrombosis is well-established, with neutrophils and platelets playing pivotal roles in the development of venous thromboembolism (VTE) [11-12]. The PLR, a novel marker that integrates information from primary hemostatic and inflammatory pathways, offers more informative value than platelet count alone. The NLR, a marker of subclinical inflammation, has been widely applied in the risk prediction and assessment of cardiovascular diseases, as it correlates with the presence and severity of coronary artery disease [13-15]. Additionally, the disruption of the blood-brain barrier (BBB) is closely related to the progression of CVT. The degradation of tight junction proteins, such as claudin-5 and occludin, affects BBB permeability, and studies have shown that plasma matrix metalloproteinase-9 (MMP-9) is associated with tight junction degradation and is closely linked to the prognosis of CVT [16-17].

With the rapid development of data science, machine learning has emerged as an advanced data analysis tool in the medical field [18]. By analyzing electronic health records, medical imaging, and genomic data, machine learning has significantly enhanced the accuracy and efficiency of disease diagnosis, particularly in discovering potential biomarkers, improving diagnostic precision, and optimizing treatment strategies [19]. For instance, machine learning can be applied in the analysis of imaging data, processing of genomic information, and the development of clinical predictive models, thereby unveiling previously unobservable patterns and providing strong support for early diagnosis, prediction of therapeutic outcomes, and personalized medicine [20]. However, the application of machine learning also faces challenges such as data quality and algorithm interpretability. Researchers need to develop new methods to improve model transparency and interpretability [21-22].

In this study, we analyzed data from 150 participants, including 100 CVT patients and 50 control subjects, to investigate the correlation between inflammatory biomarkers (such as NLR, PLR, and SII) and CVT. Multiple machine learning algorithms, including decision trees, random forests, AdaBoost, K-nearest neighbors, support vector machines (SVMs), and artificial neural networks (ANNs), were employed to enhance the diagnostic accuracy of CVT. The results demonstrated that the integration of machine learning techniques can significantly improve the diagnostic efficiency of CVT, offering new perspectives for clinical practice while providing valuable experience and guidance for medical data processing.

## Materials and methods

Study population

The study was approved by the Fuzhou 900^th^ Hospital of the People's Liberation Army (PLA) ethics committee and conducted in accordance with the Declaration of Helsinki. As it was a retrospective study, the ethics committee approved the waiver of signed informed consent, in accordance with Chinese laws and institutional requirements. This study included 150 participants, with 100 patients in the cerebral venous thrombosis group and 50 patients with primary headache serving as the control group. Inclusion criteria for the CVT group were a confirmed diagnosis of CVT based on imaging, including magnetic resonance venography (MRV), computed tomography venography (CTV), digital subtraction angiography (DSA), and availability of blood sample data upon admission. Control subjects were required to be age- and gender-matched, without CVT diagnosis or recent inflammatory conditions. The CVT group patients were diagnosed at the 900^th^ Hospital of the Joint Logistic Support Force between December 2015 and July 2022. Exclusion criteria were arterial stroke, autoimmune diseases, hematologic disorders, liver failure, inflammatory diseases, malignancies, and patients currently using anti-inflammatory, antiplatelet, or anticoagulant medications.

Data collection

We systematically collected demographic information and biochemical data from the patients, including age, sex, hypertension, diabetes, alcohol consumption, and smoking. Laboratory test results were also obtained, such as white blood cell count, platelet count, neutrophil percentage, lymphocyte percentage, monocyte percentage, and high-density lipoprotein levels. Particular attention was given to inflammatory biomarkers, such as PLR, NLR, and SII, to assess their role in CVT diagnosis. Clinical symptoms such as headache, seizures, and hemiparesthesia were recorded to explore the relationship between symptoms and inflammatory biomarkers.

Statistical analysis and machine learning model construction

This study combined statistical analysis and machine learning techniques to investigate the application and predictive value of inflammatory biomarkers in CVT diagnosis. Initial analysis was conducted using traditional statistical methods to identify key variables associated with CVT. Subsequently, various machine learning models were employed to validate the diagnostic performance of these variables, exploring the potential of data-driven predictive models to improve CVT diagnostic accuracy.

Traditional statistical analysis

All statistical analyses were conducted using Python 3.11.0 (Python Software Foundation, Fredericksburg, VA). Quantitative variables were described as mean ± standard deviation or median with interquartile range, while categorical variables were presented as counts and percentages. The Student's t-test or Mann-Whitney U test was used to analyze differences in continuous variables, and the Chi-square test was used for categorical variables. Univariate regression analysis and receiver operating characteristic (ROC) curve analysis were used to evaluate the diagnostic performance of the variables, with the optimal cut-off values determined by calculating the maximum Youden index. Due to collinearity between NLR and SII, SII and PLR were selected for multivariate analysis. A p-value of < 0.05 was considered statistically significant.

Application of machine learning

Six machine learning models were trained and tested: decision trees, random forests, AdaBoost, k-nearest neighbors, SVMs, and ANNs, to assess their performance in diagnosing CVT. These models were selected based on their widespread application in medical data analysis and their ability to handle complex data structures. Model training and testing followed a rigorous data processing protocol to ensure the reliability and validity of the results. The model's performance was evaluated using key metrics such as accuracy, recall, and F1 score. A comprehensive analysis of the results from different models was conducted to provide a scientific and accurate approach to CVT diagnosis.

## Results

General statistical data of the included patients

A total of 100 patients with CVT and 50 patients in the control group were included in this study. There were no significant differences in age distribution between the two groups (mean age 37.83 years in the CVT group vs. 39.17 years in the control group, P = 0.58), nor in the gender ratio (39.0% female in the CVT group vs. 36.0% female in the control group, P = 0.732) (Table 1). Additionally, there were no statistically significant differences between the two groups regarding comorbidities such as hypertension, diabetes, alcohol consumption, and smoking (Table 1).

**Table 1:** Baseline clinical characteristics CVT: cerebral venous thrombosis; PLR: platelet-to-lymphocyte ratio; NLR: neutrophil-to-lymphocyte ratio; SII: systemic immune-inflammatory index

Parameters	CVT group	Control group	P-value
	n = 100	n = 50	
Age (years)	37.83 ± 15.92	39.17 ± 13.24	0.580
Female (%)	39 (39.0%)	18 (36.0%)	0.732
Hypertension (%)	8 (8.0%)	8 (16.0%)	0.185
Diabetes (%)	2 (2.0%)	2 (4.0%)	0.390
Alcohol abuse (%)	6 (6.7%)	3 (5%)	0.743
Smoking (%)	8 (8.9%)	8 (13.3%)	0.427
White blood cell count (* 10⁹/L)	9.92 ± 3.42	6.93 ± 1.88	< 0.001
Platelet count (* 10⁹/L)	242 ± 80	233 ± 58	0.459
Neutrophil count (* 10⁹/L)	6.66 (4.80, 9.51)	3.81 (3.07, 4.64)	< 0.001
Lymphocyte count (* 10⁹/L)	1.59 (1.12, 2.36)	2.27 (1.78, 2.71)	< 0.001
Monocyte count ( * 10^9^/L)	0.65 ± 0.27	0.49 ± 0.14	< 0.001
High-density lipoprotein (HDL ) cholesterol (mmol/L)	1.22 ± 0.29	1.24 ± 0.33	0.667
PLR	149.52 (98.39, 198.82)	107.34 (83.31, 129.47)	< 0.001
NLR	3.93 (2.27, 7.87)	1.65 (1.31, 2.06)	< 0.001
SII	896.84(559.89, 1591.87)	382.45 (273.51, 520.92)	< 0.001

Among the CVT patients, headache was the most common symptom, occurring in 78.0% of cases, followed by isolated headache (32.0%) and seizures (24.0%) (Table 2). Hemiparesthesia was present in 22% of patients, while aphasia and abnormal mental status were less common, with rates of 4.0% and 10.0%, respectively (Table 2).

**Table 2:** The clinical symptoms of patients with cerebral venous thrombosis (CVT)

Clinical symptoms (%)	CVT group
Headache	78 (78.0)
Isolated headache	32 (32.0)
Seizures	24 (24.0)
Hemiparesthesia	22 (22.0)
Aphasia	4 (4.0)
Mental status changes	10 (10.0)
Visual-related complaints	12 (12.0)

Predictive value of inflammatory markers in CVT diagnosis

Detailed blood test analyses revealed that certain inflammation-related indicators were significantly higher in the CVT patient group compared to the control group. Specifically, the white blood cell count was significantly higher in the CVT group (9.92 vs. 6.93, P < 0.001), as was the neutrophil count (median 6.66 vs. 3.81, P < 0.001). In addition, the PLR, NLR, and SII were significantly elevated in the CVT group, indicating a higher level of systemic inflammatory response in CVT patients.

**Figure 1:**
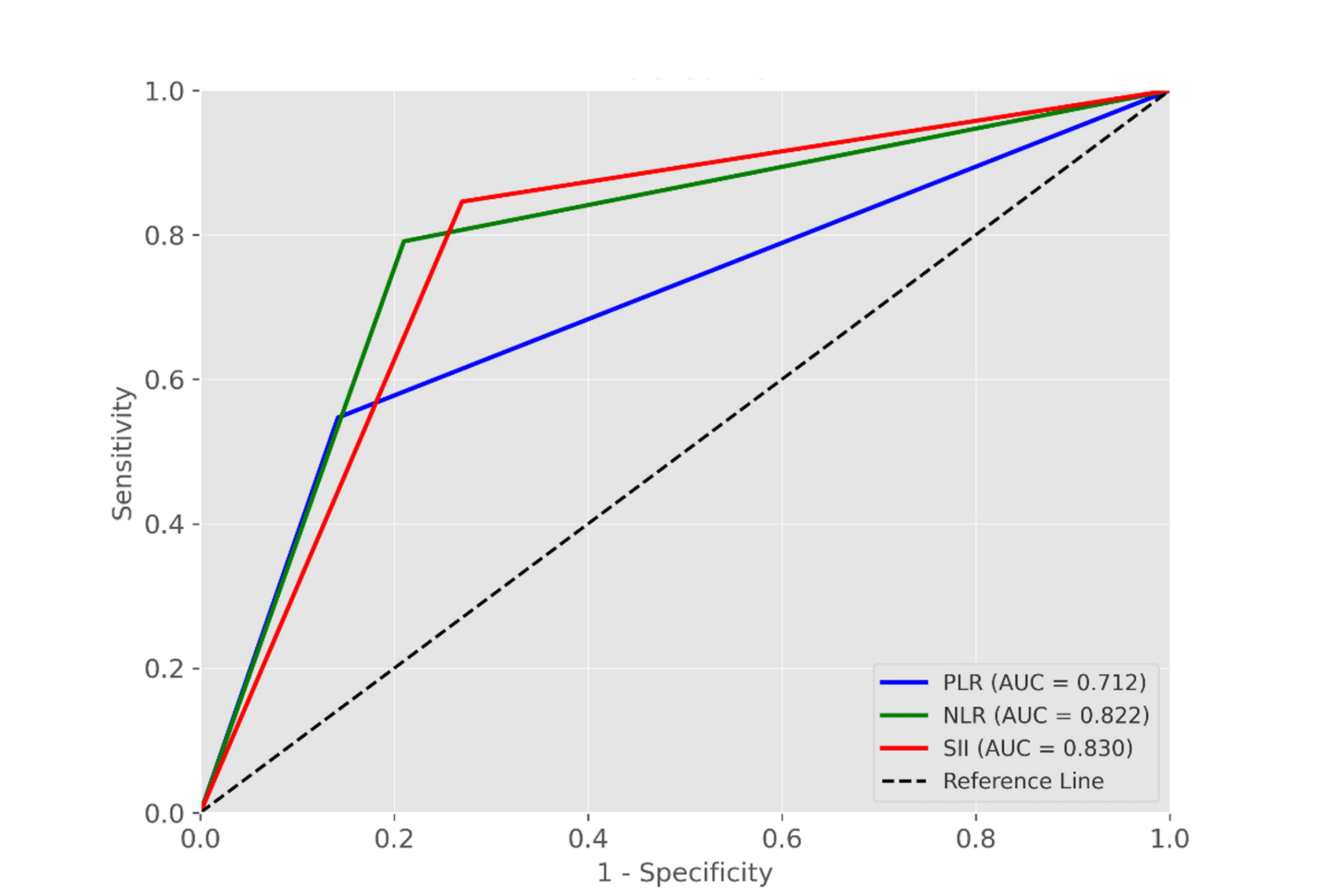
The ROC curves of NLR, PLR, and SII in the diagnosis of cerebral venous sinus thrombosis PLR: platelet-to-lymphocyte ratio; NLR: neutrophil-to-lymphocyte ratio; SII: systemic immune-inflammatory index; ROC: receiver operating characteristic curve analysis; AUC: area under the curve

The ROC curve analysis demonstrated that PLR, NLR, and SII had high predictive value in the diagnosis of CVT. The cut-off value for PLR was 145.11, with a sensitivity of 0.547, a specificity of 0.858, and an area under the curve (AUC) of 0.712 (95% confidence interval: 0.614-0.782, P < 0.001) (Figure 1). The cut-off value for NLR was 2.54, with a sensitivity of 0.791, a specificity of 0.79, and an AUC of 0.822 (95% confidence interval: 0.758-0.897, P < 0.001) (Figure 1). The cut-off value for SII was 497.05, with a sensitivity of 0.846, a specificity of 0.73, and an AUC of 0.830 (95% confidence interval: 0.754-0.896, P < 0.001) (Figure 1). These results suggest that both NLR and SII have good predictive performance in distinguishing CVT patients from non-CVT patients.

Multivariate logistic regression analysis based on SII and PLR cut-off values

**Figure 2:**
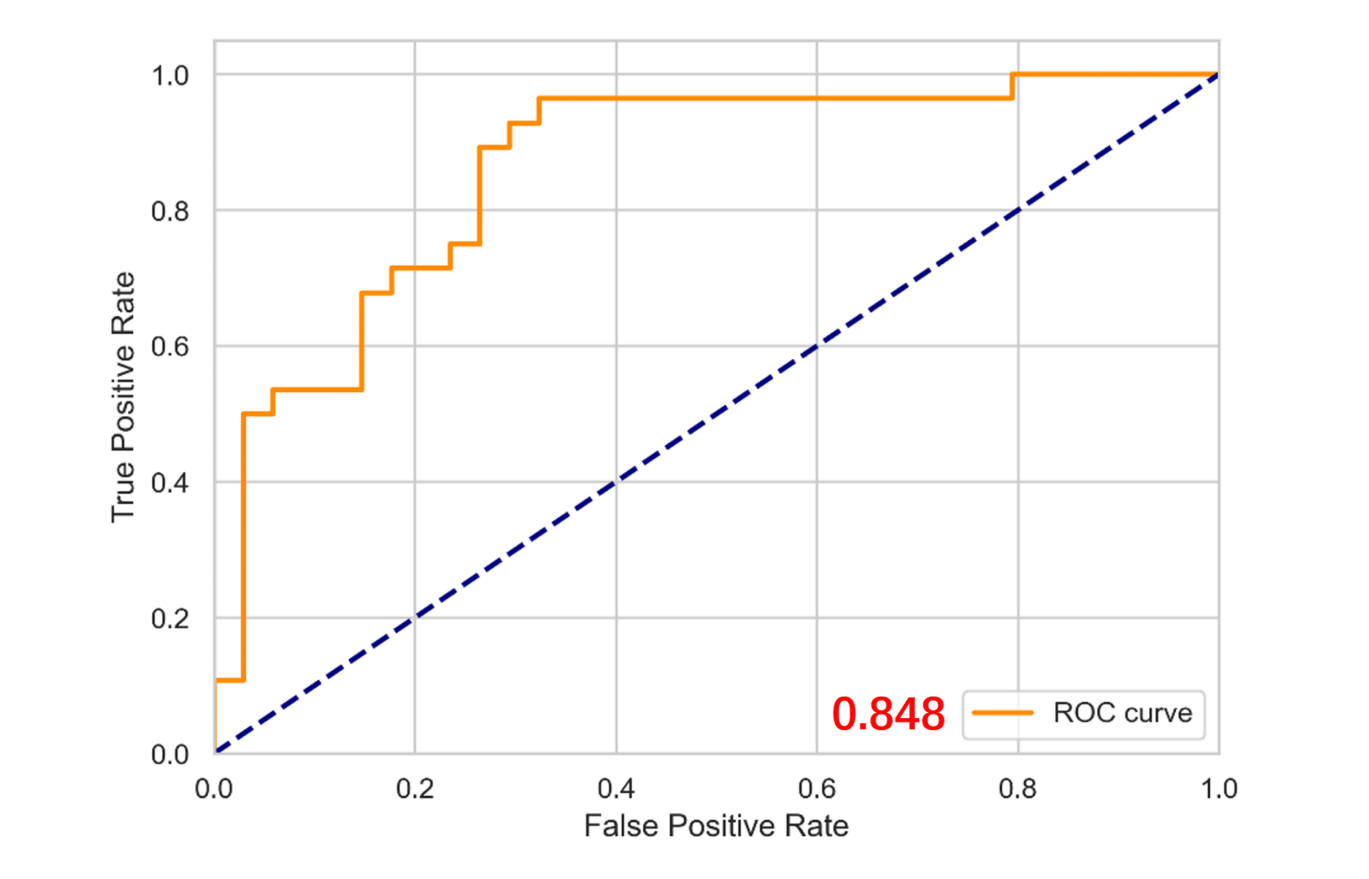
The ROC curves of PLR and SII in the diagnosis of cerebral venous sinus thrombosis ROC: receiver operating characteristic curve analysis; true positive rate (TPR): sensitivity or recall, representing the proportion of actual positive samples correctly identified as positive; false positive rate (FPR): representing the proportion of actual negative samples incorrectly identified as positive; PLR: platelet-to-lymphocyte ratio; SII: systemic immune-inflammatory index

In the multivariate logistic regression analysis, a high SII value (> 497.05) was significantly associated with the diagnosis of CVT, with an adjusted odds ratio (OR) of 13.231 (95% confidence interval: 5.479-30.312, P < 0.001) (Table 1). A high PLR value (> 145.11) also showed significant predictive value, with an adjusted OR of 2.730 (95% confidence interval: 1.124-6.152, P = 0.007) (Table 3). The regression model combining SII and PLR was evaluated using ROC curve analysis, with an AUC of 0.848 (Figure 2), indicating high diagnostic accuracy.

**Table 3:** Accuracy of inflammatory markers in CVT diagnosis PLR: platelet-to-lymphocyte ratio; NLR: neutrophil-to-lymphocyte ratio; SII: systemic immune-inflammatory index; CVT: cerebral venous thrombosis

	Cut-off value	Sensitivity	Specificity	Area under the curve (AUC)	95% Confidence Interval (AUC)	p-value
PLR	145.11	0.547	0.858	0.712	0.614-0.782	< 0.001
NLR	2.54	0.791	0.790	0.822	0.758-0.897	< 0.001
SII	497.05	0.846	0.730	0.830	0.754-0.896	< 0.001

Performance evaluation of machine learning models based on CVT inflammatory markers

**Figure 3:**
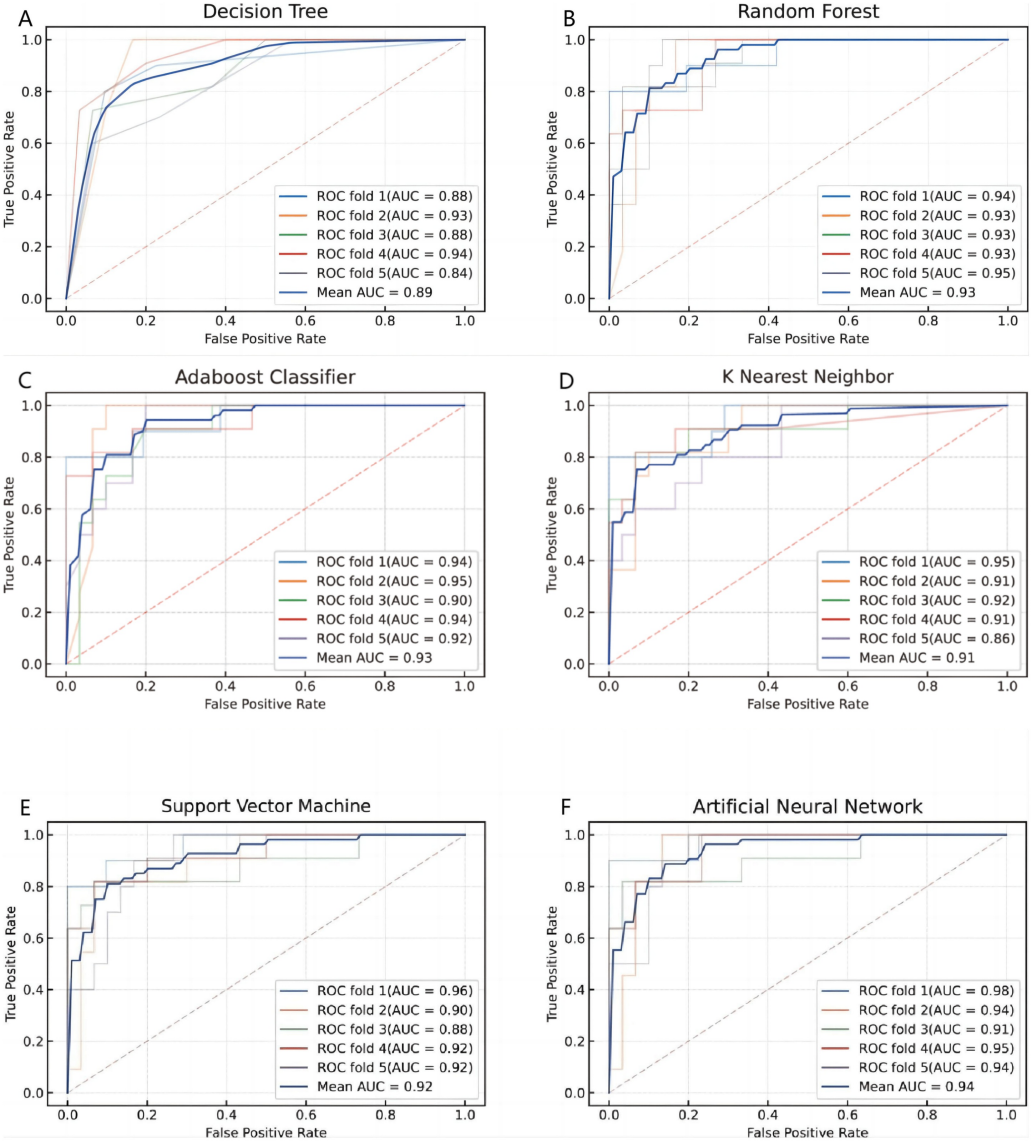
Five-fold cross-validation ROC curve results of different predictive models Figures A–F represent the decision tree, random forest, AdaBoost, K-nearest neighbors (k-NN), support vector machines (SVM), and artificial neural networks (ANN), respectively. ROC: receiver operating characteristic curve analysis; AUC: area under the curve

**Figure 4:**
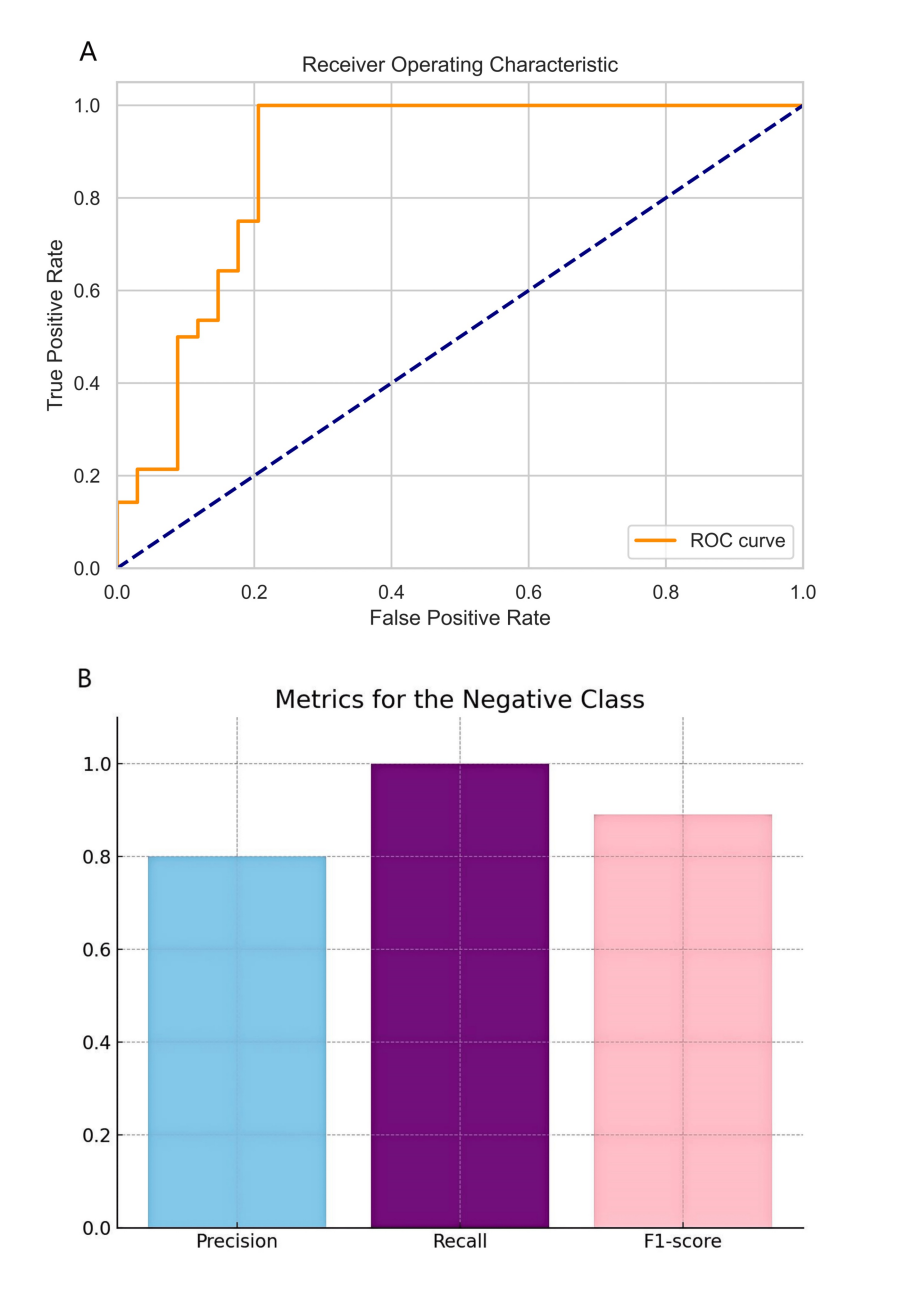
The ROC curve and model evaluation of the artificial neural network (ANN) model Panel A shows the average AUC value after the five-fold cross-validation of the ANN. Panel B shows the accuracy, recall, and F1 score. ROC: receiver operating characteristic curve analysis

The ANN model based on SII and PLR showed the best performance in diagnosing CVT, with an average ROC value of 0.94 in 5-fold cross-validation (Figures 3A-3F), indicating high classification performance. Additionally, the ANN model achieved an accuracy of 0.8, a recall of 0.985, and an F1 score of 0.91 (Figures 4A-4B), demonstrating the model's high predictive accuracy and reliability in CVT diagnosis. The high recall rate helps minimize missed diagnoses, while the high F1 score reflects a good balance between precision and recall.

## Discussion

This study focuses on analyzing inflammatory biomarkers, particularly the SII and NLR, for their potential value in the early diagnosis of CVT. Through the analysis of blood samples from 150 patients, it was found that CVT patients had significantly higher levels of SII and NLR compared to the control group, with an average SII value of 896.84 and an average PLR of 149.52, which were considerably elevated compared to healthy controls. These findings highlight the importance of inflammatory markers in identifying CVT and provide clinicians with new diagnostic tools.

The study also documented the clinical manifestations of CVT patients in detail, finding that headache was the most common symptom, reported by 78 patients (78.0%), with 32 patients (32.0%) presenting with isolated headaches without other neurological symptoms. This suggests that headache may be a key symptom in the early diagnosis of CVT, particularly in the absence of other evident neurological signs [23-24]. For patients presenting with an isolated headache, especially if the headache's nature has changed or worsened, the possibility of CVT should be considered.

The diverse and nonspecific clinical presentation of CVT contributes to delayed or missed diagnoses. This study emphasizes the potential of inflammatory biomarkers, such as SII and NLR, in reducing misdiagnosis rates and improving diagnostic efficiency. A combined assessment of clinical symptoms (such as headache characteristics) and biomarkers (such as SII and NLR levels) may provide a more effective and accurate method for early identification and treatment of CVT, especially as a screening tool in primary healthcare settings to determine which patients require further specialized diagnostic evaluation and treatment. The ROC curve analysis confirmed the predictive capabilities of PLR and SII in CVT diagnosis, demonstrating high diagnostic accuracy.

The pathogenesis of cerebral venous sinus thrombosis can be traced back to Virchow's triad, which includes vascular wall injury, hypercoagulability, and blood stasis [25-27]. Inflammation and infection are known risk factors that promote thrombosis [28]. The SII, which combines platelet count, neutrophil count, and lymphocyte count, reflects both inflammation and thrombosis states, making it more stable and representative compared to other markers [29].

Both PLR and SII, as reliable indicators of thrombosis and inflammation, have recently been identified as prognostic markers for various cardiovascular and cerebrovascular diseases [12]. In the context of CVT, elevated PLR and SII may reflect the complex interplay between inflammatory and coagulation activities [28]. The SII not only provides information about the patient's inflammatory status but also may indicate the severity of thrombus burden [29]. For patients with unexplained headaches and normal CT scans, clinicians should be vigilant about the possibility of CVT and consider further imaging evaluation (e.g., MRI/MRV) to shorten the diagnostic timeframe and improve patient outcomes.

The application of machine learning in healthcare is becoming increasingly widespread, providing new methods for disease diagnosis and treatment. In this study, six supervised learning models were constructed and evaluated based on SII and PLR, including decision trees, random forests, AdaBoost, k-nearest neighbors, SVM, and ANN, to enhance the diagnostic accuracy of CVT. Notably, the ANN model performed the best, with a ROC value of 0.94, and achieved high levels of accuracy, recall, and F1 score, demonstrating its great potential for CVT diagnosis.

To ensure the clinical effectiveness of machine learning models, appropriate validation and measures to prevent overfitting are essential. By using five-fold cross-validation and multiple metrics for evaluation, this study minimized the risk of overfitting and ensured the generalizability of the models. However, before applying these models in real clinical settings, further validation in a broader patient population is required.

This study has several limitations. First, the sample size was relatively small, which may limit the generalizability of the findings. Second, the study was conducted in a single center, which may introduce selection bias. Third, the use of machine learning models requires further validation in broader and more diverse populations to ensure their clinical applicability. Finally, the cross-sectional design limits the ability to establish causal relationships between inflammatory biomarkers and CVT.

## Conclusions

This study demonstrates that inflammatory markers such as PLR, NLR, and SII have significant predictive value in the diagnosis of CVT. SII, in particular, showed outstanding diagnostic efficacy through ROC curve analysis. In addition, machine learning models, especially ANN, have great potential to improve diagnostic efficiency and accuracy, with the ANN model achieving the best performance. Future studies with larger sample sizes and prospective designs are needed to further validate the clinical application of these markers and models, optimizing the CVT diagnostic workflow.
